# Significance of segmental glomerulosclerosis in IgA nephropathy: What is the evidence?

**DOI:** 10.12861/jrip.2013.36

**Published:** 2013-10-10

**Authors:** Muhammed Mubarak, Hamid Nasri

**Affiliations:** ^1^Department of Histopathology, Sindh Institute of Urology and Transplantation (SIUT), Karachi, Pakistan; ^2^Department of Nephrology, Division of Nephropathology, Isfahan University of Medical Sciences, Isfahan, Iran

**Keywords:** IgA nephropathy, Focal segmental glomerulosclerosis, Oxford classification, Columbia classification, Proteinuria, Renal failure

**Implication for health policy/practice/research/medical education:**

Primary IgA nephropathy (IgAN) is the commonest glomerulopathy worldwide. Its clinical presentation, histology and prognosis vary widely. The lesions of segmental glomerulosclerosis are common in IgAN and represent participation of at least three pathogenetic mechanisms. It is important to identify the different subtypes of segmental sclerosing lesions as these are of prognostic value.

Primary IgA nephropathy (IgAN) is the commonest primary glomerular disease worldwide (1,2). The disease is notorious for a wide variation in the epidemiology, clinical presentation, histology, and the prognosis (3,4). The only unifying feature is the presence of dominant or co-dominant IgA deposits in the mesangium and occasionally in the peripheral capillary loops on immunofluorescence (IF) microscopy (5-9). The reported frequency ranges from 2 to 52% of all renal diseases in renal biopsy series from different parts of the world (1,2). The clinical presentation ranges from asymptomatic to acute renal failure to end-stage renal disease (ESRD). The typical pathological lesion is the mesangial proliferative glomerulonephritis (MesPGN), but the spectrum of pathological lesions is broad and the morphological lesions of segmental glomerulosclerosis are common on renal biopsies in patients with primary IgAN (10-16).

Hill *et al*. noted in their preliminary analysis the presence of such lesions in 66% of cases (17). In a subsequent detailed study, some form of segmental glomerulosclerosis was found in 78.9% of biopsies (18,19). The original study cohort of the Oxford classification showed such lesions in 76% of the biopsies (10). We also found the lesions of segmental glomerulosclerosis in 63.2% in a cohort of consecutive cases of primary IgAN (unpublished data). The exact origin and pathogenesis of these lesions is still enigmatic. It is possible that, there are at least three ways by which segmental glomerulosclerosis may occur in patients with IgAN. First; by post-inflammatory scarring of segmental proliferative or necrotizing lesions, commonly observed in this disease, second; due to compensatory hemodynamic changes following nephron loss, and finally by primary podocyte damage, perhaps due to mediators released from mesangial cells (18,19) or perhaps due to direct cytotoxic action of IgA1 deposits. This schema of events is shown schematically in Figure 1.

**Figure 1:**
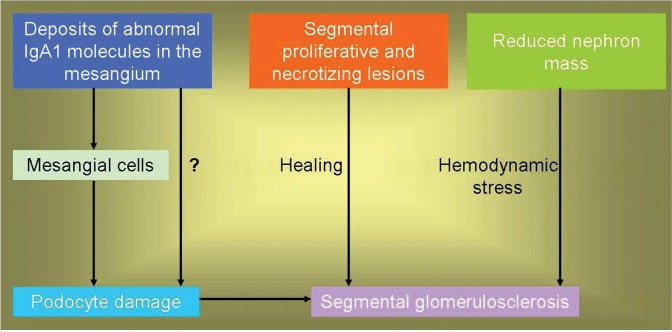
Schematic diagram showing the different possible pathways of origin of segmental glomerulosclerosis in IgA nephropathy (IgAN)

We and some other investigators are of the view that it is possible to identify two types of segmental sclerosing lesions on renal biopsy examination in these patients (17,18). First is the focal segmental glomerulosclerosis (FSGS), which is classified as a separate category in the Hass classification of IgAN and more recently defined more elaborately in Columbia classification (20,21). This lesion may be considered as “definite FSGS” lesion or the FSGS of Columbia classification and is the result of podocyte injury. How the podocyte injury is induced by IgA deposits is not known at preset. The second mechanism involves fibrosis or healing process arising on the background of segmental proliferative and necrotizing lesions. This may be considered the “indeterminate form” of segmental glomerulosclerosis or FSGS of Oxford classification. The prognosis of these types of segmental lesions is different as observed by Hill *et al*. who noted that patients with frank FSGS had a prognosis, with 50% ending on dialysis, compared with 4.1% of those without such lesions. Patients with indeterminate segmental lesions had a prognosis intermediate between the other two groups, with 36.1% ending on dialysis (17). However, this issue is still far from completely settled (22-24).

Regarding the pathological features distinguishing between these two types of segmental lesions, Hill *et al*. suggested that intracapillary hyalinosis and prominent podocyte hypertrophy/hyperplasia favor the diagnosis of definite FSGS over indeterminate segmental glomerulosclerosis (17,18). The pathological criteria for the diagnosis of Oxford segmental lesion are less stringent and require the mere presence of segmental increase in mesangial matrix with obliteration of capillary lumena with or without capsular adhesions as sufficient criterion for the diagnosis of segmental sclerosis (10). In contrast, the Columbia classification requires additional features such as intracapillary hyalinosis, foam cells, podocyte alterations and intracapillary cellularity to define the five different types of FSGS lesions (21,24).

We also take this opportunity to emphasize the fact that it is possible to distinguish objectively and reproducibly between the S variable of Oxford classification and the FSGS of the Columbia classification. As discussed earlier, the lesions of podocyte hypertrophy/hyperplasia and intracapillary hyalinosis favor the later over former and these should be looked for carefully when examining renal biopsies from patients with IgAN with segmental glomerular sclerotic lesions. We additionally hypothesize that S variable of Oxford classification represents an early stage in the progression of glomerular lesions in IgAN and the FSGS lesion, the more advanced stage with injury to the podocyte compartment of the glomeruli, which finally culminates in the diffuse chronic sclerosing glomerulonephritis (GN) and end-stage renal disease (ESRD). We believe that the lesions of FSGS, as defined by Columbia classification, represent an advanced morphological stage of progression in IgAN and do not represent idiopathic FSGS superimposed on IgAN as two separate but concurrent diseases.

In conclusion, the presence of FSGS-like lesions in the background of IgAN is associated with the poor clinical and pathological prognostic factors and outcome of this disease and the lesions are of prognostic significance. Two types of segmental sclerosing lesions can be identified on careful renal biopsy examination and an attempt should be made to subclassify them as these have vastly different prognosis.

## Authors’ contributions

MM wrote the paper, drafted the paper and gave final approval. HN provided intellectual input and help in preparing the paper.

## Conflict of interests

The author declared no competing interests.

## Ethical considerations

Ethical issues (including plagiarism, misconduct, data fabrication, falsification, double publication or submission, redundancy) have been completely observed by the authors.

## Funding/Support

None.
